# *Radix Astragali* Stimulates p38 MARK Phosphorylation in Pediatric Patients with *β*-Thalassemia

**DOI:** 10.1155/2016/7468979

**Published:** 2016-11-02

**Authors:** Zhuoming Lu, Xinhua Qian, Chunhong Zhang, Zhiwen Chen, Guangliang Du

**Affiliations:** ^1^Guangzhou Hospital of Traditional Chinese Medicine, Guangzhou 510130, China; ^2^Nanfang Hospital, Southern Medical University, Guangzhou 510515, China

## Abstract

A previous study conducted by our group demonstrated that* Radix Astragali* compounded with* Codonopsis pilosula* and* Plastrum testudinis* was effective in treating pediatric *β*-thalassemia in a randomized, controlled clinical trial. However, the mechanism of action that underpins this treatment remains to be elucidated. Blood was collected from patients participating in this clinical trial and nucleated red blood cell-enriched mononuclear cells were isolated to facilitate the extraction of RNA and protein. RT-PCR was used to monitor the expression of globin genes and p38 MAPK, and total and phosphorylated p38 MAPK expression was assessed using Western blot analysis. Expression of *α*-, *β*-, and A*γ*-globin mRNAs was not significantly affected following treatment with* R. Astragali* or the compounded formulation. However, G*γ*-globin mRNA levels increased significantly in both treatment groups (when compared with pretreatment levels) following 12 weeks of treatment. Moreover, posttreatment G*γ*-globin expression was significantly higher in both treatment groups compared with the control group. Although neither p38 MAPK mRNA nor protein levels were affected by the treatments, posttreatment phosphorylation of p38 MAPK was significantly increased in the* R. Astragali* and compounded formulation groups compared with the control group. These data suggest that the molecular mechanisms that underpin the efficacious use of* R. Astragali* (and its compounded formulation) in pediatric *β*-thalassemia treatment facilitate the induction of G*γ*-globin expression following activation of p38 MAPK.

## 1. Introduction

Hemoglobin (Hb) disorders, particularly *β*-thalassemia, are the most common single gene disorders caused by mutations in the *β*-globin locus. Resultant disorders cause abnormal or reduced adult hemoglobin (HbA) production and excess, unmatched *α*-chains in developing erythroblasts. Clinical symptoms include bone marrow expansion, splenomegaly, and a severe anemia that requires regular blood transfusions. Clinical management of *β*-thalassemia patients includes lifelong blood transfusions and chelation therapy to remove excess transfused iron [1] and, in some cases, bone marrow transplantation [2, 3]. *β*-Thalassemia syndromes are classified according to severity based on steady-state Hb and transfusion dependency. *β*-Thalassemia major and *β*-thalassemia intermedia are both caused by the inheritance of two *β*-globin gene mutations [4]. Furthermore, *β*-thalassemia results in moderate anemia in childhood, which often progresses to transfusion dependency over time, iron loading, and unique complications related to expanded erythropoiesis and hemolysis.

Although allogeneic hematopoietic stem cell transplantation (HSCT) and gene transfer therapy can be used for patients with *β*-thalassemia, modern medicines and the requisite financial resources for these procedures are not available to most *β*-hemoglobinopathy patients [5–7]. The pharmacological induction of fetal hemoglobin (HbF) may be a promising alternative therapeutic option for treatment of these patients. HbF is comprised of two *α*-globin and two *γ*-globin chains (*α*_2_ *γ*_2_) and is highly expressed during fetal and early postnatal periods; however, HbF levels subsequently decline following concurrent increases in HbA (*α*_2_ *β*_2_) expression. An increase in HbF levels can functionally compensate for the shortfall in HbA synthesis in *β*-thalassemia patients and synthesized *γ*-chains can neutralize excess unbalanced *α*-chains to reduce erythrocyte damage and ameliorate anemia and many of the complications associated with *β*-thalassemia [5]. Pharmacological induction of HbF also represents a potentially less expensive and more widely applicable means to treat *β*-hemoglobinopathies worldwide. Drugs that facilitate the induction of *γ*-globin include hydroxyurea (HU), 5-azacytidine, butyrate, and erythropoietin, among which HU is the only candidate that has been proven to improve the clinical course of *β*-thalassemia [8–11]. However, these agents are unsuitable in relation to efficacy, safety, and ease of use. For example, responses to HU are variable and weakened responses are often observed following long-term administration. In addition, *γ*-globin induction agents may cause myelotoxicity and are potential carcinogens. Furthermore, the cost of these treatment regimens is a significant limitation [7]. Thus, effective and safe alternative gene induction reagents are highly desirable [12–14].

Traditional Chinese medicine (TCM), alone or in combination with other treatment regimens, has been utilized to treat anemia [2, 15–17]. It has been suggested that Yisui shengxue granules [18] and* Plastrum testudinis* (tortoise plastron) [19] prolong erythrocyte lifespans via antilipid peroxidation and membrane stabilization.* Astragalus membranaceus* (*Radix Astragali* [AMW]; “Huang Qi” in Chinese) roots have previously been used in TCM to treat anemia [16, 20]. Our previous* in vitro* experiments indicated that* Plastrum testudinis*, AMW, and* Codonopsis pilosula* could enhance *γ*-globin mRNA expression and HbF production in both human erythroid K562 cell cultures and erythroid progenitors from patients with *β*-thalassemia [19, 21]. Subsequently, we conducted a randomized, controlled, double-blind clinical trial with AMW and its compounded formulation (AMW +* Codonopsis pilosula* +* Plastrum testudinis*) to treat *β*-thalassemia [14]. The resultant data indicated that, following 12 weeks of treatment, AMW and the compounded formulation increased the level of HbF, erythrocytes, mean corpuscular Hb, and reticulocytes in *β*-thalassemia, with no reported side effects [14]. Thus, AMW and the compounded formulation can ameliorate *β*-thalassemia symptoms by inducing HbF gene expression. Furthermore, an additional study performed by our group demonstrated that* Radix Astragali* and the compounded formulation can independently induce p38 phosphorylation in K562 cells and cultured human erythroid progenitor cells from the peripheral blood of *β*-thalassemia patients [22]. However, these results require further validation. Thus, we aim to elucidate the mechanism of action pertaining to AMW and its compounded formulation in *β*-thalassemia patients, with studies focusing on globin gene expression and downstream signal pathways.

## 2. Materials and Methods

Research in this study was performed in accordance with the Declaration of Helsinki and the study was approved by the Ethics Committee of Guangzhou Hospital of Traditional Chinese Medicine. All participating patients or their legal guardians signed informed consent forms before initiating the study.

### 2.1. Samples

The blood samples that were utilized in this study were from participants of a previous randomized, controlled, double-blinded clinical trial [14]. The trial included 35 patients, who were between two and 18 years of age, with confirmed *β*-thalassemia based on the* Criteria for Diagnosis and Treatment of Hematopathy* [23]. The subjects had not received a blood transfusion or treatment for anemia in the previous 12 weeks prior to enrollment and associated hemoglobin levels ranged from 4.5 to 10 g/dL at the time of enrollment. Thirteen of the subjects were treated with* R. Astragali* (AMW), 11 patients were treated with the compounded formulation (AMW +* Codonopsis pilosula* granule [CPG] +* Plastrum testudinis* granule [PTG]), and 11 patients were treated with a placebo. Pilot experiments performed in our laboratory (data not shown) indicated that a sample size greater than 10 subjects per group was required for this analysis.

Peripheral blood was drawn from the patients before treatment and 12 weeks after treatment as designated in the clinical trial [14]. All of the patients (or their legal guardians) permitted us to use the blood samples that were extracted in the present study to study the molecular mechanisms underlying the treatment effects. Blood samples were collected into whole-blood tubes containing Na-EDTA and aliquots from these samples were used for this study.

### 2.2. Treatment

The treatment procedure and information pertaining to the effectiveness of treatments with AMW and the compounded formulation were previously reported by Lu et al. (2012) [14]. Briefly, AMW and the compounded formulation (AMW + CPG + PTG) were produced by Guangdong Yifang Pharmaceutical Co. Ltd. (Guangdong Province, China). Herbs were extracted and granulated with 10 g of* R. Astragali* per g of AMW, 10 g of* Codonopsis pilosula* in 3 g of CPG, and 10 g of* Plastrum testudinis* in 0.7 g of PTG. The placebo was prepared using dextrin and an identical protocol to that used for herb granulation. All granules were administered orally with warm water in the fasted state and treatment was administered for 12 weeks.

### 2.3. Peripheral Blood Mononuclear Cell Isolation

A mononuclear cell population that consists mostly of nucleated red blood cells was isolated from peripheral blood using discontinuous Percoll density gradient centrifugation. Two milliliters of peripheral blood from each of the pediatric patients was placed in a centrifuge tube. Each blood aliquot was subsequently diluted with 2 mL of PBS (pH 7.4). A double-density Percoll column was prepared with one milliliter each of 1.090 g/mL and 1.075 g/mL diluted Percoll cell isolation media (Pharmacia). The 1.090 g/mL Percoll solution was layered at the bottom of a centrifuge tube, and the 1.075 g/mL Percoll solution was slowly and gently dispensed from a syringe over the denser solution without disturbing the interface. Four milliliters of diluted peripheral blood sample was slowly added to the top of the double-density Percoll column and centrifuged at room temperature at 400 rpm for 30 min. The cell layer between the 1.090 g/mL and 1.075 g/mL Percoll media was isolated. This cell population contained mostly nucleated red blood cells and a small number of lymphocytes and granulocytes [24]. These cells were washed three times with two volumes of PBS. This was followed by a centrifugation step at 1,250 rpm for 10 min to discard the supernatant and keep the pellet. These cells were used for RNA and protein extraction.

### 2.4. Measurement of Globin mRNA

RNA was isolated from nucleated red blood cell-enriched mononuclear cells using TRIzol® reagent (Sangon Biotech, Shanghai, China) according to the manufacturer's instructions. One hundred units of M-MLV (Invitrogen, Carlsbad, CA) were used to synthesize cDNA from 4 *μ*L of total RNA. Quantitative real-time PCR was then performed using SYBR Green PCR Master Mix (Applied Biosystems, Norwalk, CT) and an ABI 3900 real-time system (Applied Biosystems). The following primer and probe sequences were used: *α*-globin forward primer (5′-GAGGCCCTGGAGAGGATGTT-3′) and *α*-globin reverse primer (5′-CGTGGCTCAGGTCGAAGTG-3′); *β*-globin forward primer (5′-GGCAACCCTAAGGTGAAGGC-3′) and *β*-globin reverse primer (5′-GCAGCTCACTCAGTGTGGCA-3′); A*γ*-globin forward primer (5′-GAGCTCACTGCCCATGAAT-3′) and A*γ*-globin reverse primer (5′-CTCTCAGCAGAATAGATTTATTATTTCT-3′); G*γ*-globin forward primer (5′-AGCTCACTGCCCATGAAG-3′) and G*γ*-globin reverse primer (5′-CTCTTAGCAGAATAGATTTATTATTTCA-3′); and p38 MAPK forward primer (5′-ACGTTCTACCGGCAGGAGCT-3′) and p38 MAPK reverse primer (5′-AAGCAGCACACACAGAGCCA-3′). Human *β*-actin was used as an endogenous control; the forward primer sequence used for the amplification of this locus was 5′-GCGCGGCTACAGCTT CA-3′ and the reverse primer sequence was 5′-TCTCCTTAATGTCACGCACGAT-3′. All data were analyzed after normalization to *β*-actin expression and each reaction was performed in triplicate. RT-PCR experiments were performed in triplicate.

### 2.5. Preparation of Standard cDNA and Standard Curve

Three cDNA samples were randomly selected from each group and amplified using the corresponding primer pair to prepare standard control DNA. PCR products were separated and extracted from agarose gels. DNA concentration was measured using spectrophotometry (260 nm). DNA was considered to be of high quality if the OD_260 nm_/OD_280 nm_ ratio was between 1.8 and 2.0.

mRNA was measured following 10-fold serial dilution of standard DNA (10^8^ to 10^4^ copies in ultrapure water). This determination was performed in triplicate following the use of a SYBR Green real-time RT-PCR assay. Associated Ct values were plotted against DNA copy number to construct a standard curve.

### 2.6. SYBR Green Real-Time RT-PCR Assay

A real-time RT-PCR assay was carried out using the SYBR Green Master Mix (Invitrogen) Kit. A concentration of 0.2 *μ*M of each primer was used in each reaction. RT-PCR was performed in a final volume of 25 *μ*L with each reaction containing 5 *μ*L of 5 × SYBR Green Master Mix, 0.5 *μ*L of each of 10 *μ*M forward and reverse primers, 0.5 *μ*L of dNTPs (10 mmol/L), 2 *μ*L of cDNA, 0.5 *μ*L of Taq (3 U/*μ*L), and 16.5 *μ*L of ultrapure water. The optimized thermal cycling conditions were as follows: 40 cycles of 93°C for 3 min, 93°C for 15 s, 55°C for 25 s, and 72°C for 25 s. Fluorescence was measured at the end of each cycle.

### 2.7. Western Blot and Antibodies

For whole-cell extract preparation, nucleated red blood cell-enriched mononuclear cells were lysed in radioimmunoprecipitation assay (RIPA) buffer containing a complete protease inhibitor cocktail (Roche, Penzberg, Upper Bavaria, Germany) for 10 min at 4°C and the extract (50 *μ*g) was separated using SDS-PAGE and transferred onto polyvinyl difluoride (PVDF) membranes. Membranes were incubated with rabbit anti-p38 MAPK antibody (1 : 500), goat anti-mouse *β*-actin antibody (Santa Cruz Biotechnology, Santa Cruz, CA), or rabbit anti-phospho-p38 MAPK antibody (1 : 500, Bioworld Technology, St. Louis Park, MN). HRP-conjugated anti-goat or anti-rabbit secondary antibodies (both 1 : 5000) were purchased from Sigma Aldrich (St. Louis, MO) and Santa Cruz Biotechnology, respectively. The sample sizes pertaining to the Western blot analyses for the three groups were similar to those described for previous experiments; that is, 13 patients treated with* R. Astragali*, 11 patients treated with the compounded formulation, and 11 patients treated with the placebo drug were analyzed during the Western blot analyses.

### 2.8. Statistical Methods

Statistical analysis was conducted using SPSS software (Version 13.0). Results are presented as means ± standard deviation (SD). Greenhouse-Geisser corrections for nonsphericity were taken into account when determining significance. Student's* t*-test or one-way ANOVA was used to assess statistical significance among mean values for groups. Following ANOVA analysis, Fisher's Least Significant Difference (LSD) test was performed to confirm data and compare means (*p* ≤ 0.05 was considered statistically significant).

## 3. Results and Discussion

### 3.1. Standard Curves and Assay Reliability

A mononuclear cell population that consists mostly of nucleated red blood cells was harvested from patients and RNA was subsequently extracted. For each sample, *γ*- and *β*-globin mRNA levels were measured using a fluorescence-based RT-PCR assay to analyze the molecular mechanisms associated with 12 weeks of AMW and compounded formulation treatment. Ct values were plotted against diluted standard DNA copy number and the associated standard curve displayed a linear range across 3-4 log units of DNA copy number (10^8^–10^4^ copies/*μ*L for G*γ*-globin; 10^9^–10^5^ copies/*μ*L for *α*-, *β*-, and A*γ*-globin; and 10^9^ to 10^6^ copies/*μ*L for p38 MAPK). Linear regression analysis was used to establish standard curves for *α*-globin (*y* = −3.047*x* + 36.290; *R* ^2^ = 0.999), *β*-globin (*y* = −3.008*x* + 36.606; *R* ^2^ = 0.999), G*γ*-globin (*y* = −3.128*x* + 35.796; *R* ^2^ = 0.999), A*γ*-globin (*y* = −3.438*x* + 39.598; *R* ^2^ = 0.990), p38MAPR (*y* = −3.082*x* + 36.240; *R* ^2^ = 0.999), and *β*-actin (*y* = −3.394*x* + 39.996; *R* ^2^ = 1.000).

### 3.2. G*γ*-Globin mRNA before and after Treatment

**Figure 1:**
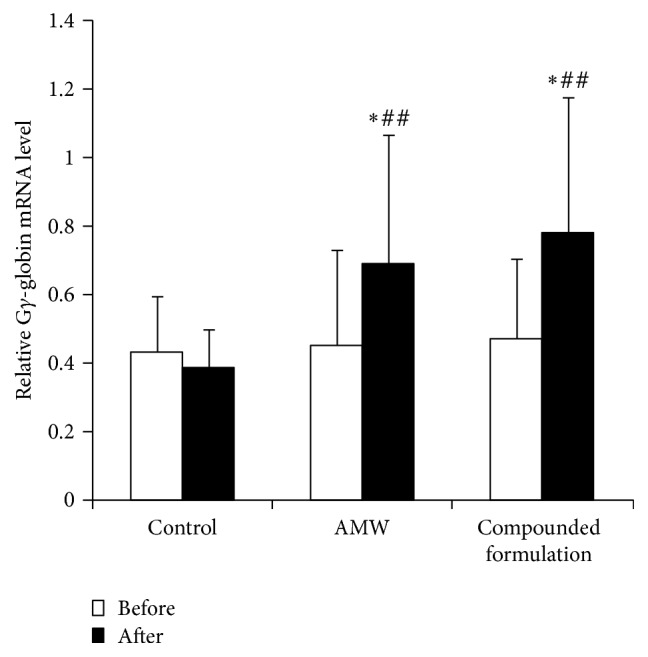
Relative G*γ*-globin gene expression before and after treatment of pediatric *β*-thalassemia. ^*∗*^ *p* < 0.05 compared with the corresponding control; ^##^ *p* < 0.01 compared with before treatment of the same group.

**Figure 2:**
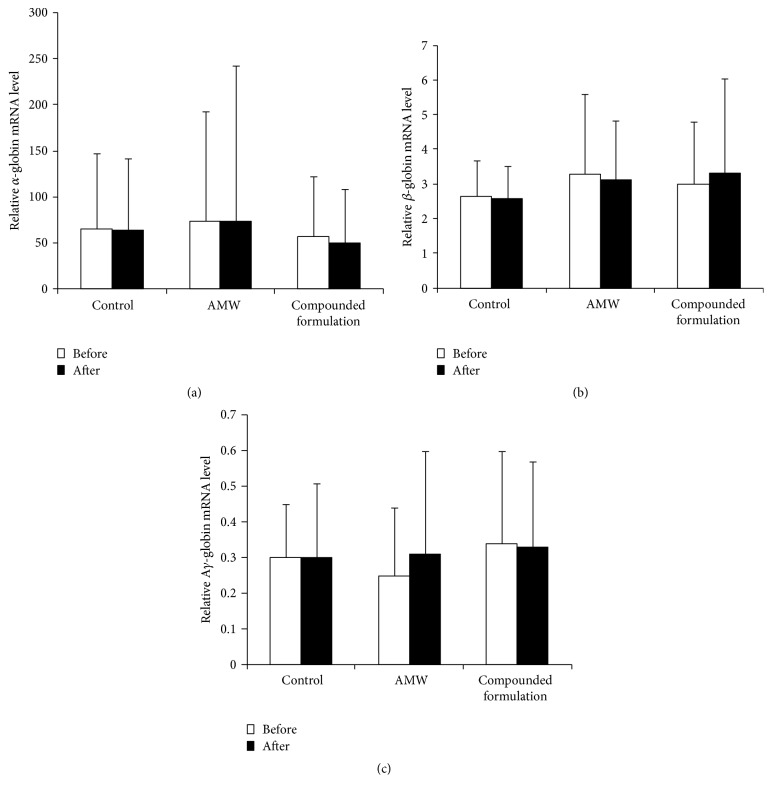
Relative globin gene expression before and after treatment of pediatric *β*-thalassemia: (a) *α*-globin; (b) *β*-globin; and (c) A*γ*-globin.

Cells express *α*-, *β*-, and *γ*-globin genes during fetal and adult development in order to synthesize hemoglobin. Adult hemoglobin A is a tetramer containing two *α*-globin subunits and two *β*-globin subunits (*α*_2_ *β*_2_). Conversely, fetal hemoglobin is composed of two *α*-globin and two *γ*-globin subunits. Evaluation of globin gene expression in nucleated red blood cell-enriched mononuclear cells collected from patients before and after treatment using standard RT-qPCR and primer pairs specific to human *α*-, *β*-, and *γ*-globin revealed expression of mRNA transcripts specific to these globin genes. RT-PCR data indicate that G*γ*-globin mRNA did not differ significantly between groups before treatment (*p* = 0.923) but was significantly different between groups after treatment (*p* = 0.025). Further multiple comparisons showed that G*γ*-globin mRNAs from the compounded formulation group (*p* = 0.010) and the* Radix Astragali* treatment group (*p* = 0.036) were significantly higher than the control group; no significant difference was found between the compounded formulation group and the* Radix Astragali* treatment group (*p* = 0.516). Significant differences occurred between the placebo and treatment groups; however, the difference between the AMW and the compounded formulation group was not significant (Figure 1). The G*γ*-globin mRNA levels in the compounded formulation group were 1.65-fold higher following treatment compared with levels before treatment (*p* = 0.004). Similarly, the G*γ*-globin mRNA levels in the* Radix Astragali* treatment group were 1.53-fold higher following treatment compared with levels before treatment (*p* = 0.002). These differences were deemed significant. In contrast, no significant differences were observed in G*γ*-globin levels in patients from the control group before and after placebo drug treatment (*p* = 0.293) (Figure 1), suggesting that AMW and its compounded formulation significantly increased G*γ*-globin mRNA transcription. The latter phenomenon might explain the increased HbF levels that were observed in the clinical trial. However, neither AMW nor its compounded formulation affected *α*-, *β*-, or A*γ*-globin mRNA levels after 12 weeks of treatment (Figure 2).

### 3.3. p38 MAPK Signaling Pathway Changes before and after Treatment

p38 MAPK was initially identified as a protein kinase that is activated by stress. This kinase has also been shown to coordinate cellular responses during erythropoiesis [25–27]. p38 MAPK is essential for hemoglobin synthesis and p38 mice exhibit severe anemia and die* in utero* due to defective angiogenesis and placental insufficiency [28]. In response to sodium butyrate and trichostatin A treatment of erythroid cells, p38 MAPK mediates HbF induction by activating cAMP response element binding protein 1 (CREB1). p38 signaling regulates G*γ*-globin transcription during erythroid maturation via a downstream effector, CREB1, which binds to the G*γ*-globin cAMP response element (G-CRE). Moreover, gain of p38 or CREB1 function augments *γ*-globin transcription and these regulatory effects were observed to be conserved under tested physiological conditions in primary erythroid cells [29].

Given that the p38 MAPK signaling pathway is the key mediator pertaining to stress induction of *γ*-globin mRNA [30], we proposed that the induction of G*γ*-globin gene expression following AMW and compounded formulation treatment may increase p38 MAPK expression or activation. Indeed, a previous study performed by our group demonstrated that one of the compounded agents,* Plastrum testudinis*, facilitated *γ*-globin mRNA accumulation and HbF synthesis in K562 cells following activation of the p38 MAPK signaling pathway. These results were validated following the observation that the latter effects were suppressed after pretreatment of the K562 cells with the p38 MAPK inhibitor, SB203580 [30]. Furthermore, a separate study conducted by our group demonstrated that aqueous extracts of* Radix Astragali*, aqueous extracts of the compounded formulation, and 0.5 mmol/L sodium butyrate all independently induced p38 phosphorylation in K562 cells and cultured human erythroid progenitor cells from the peripheral blood of *β*-thalassemia. All of these reactions demonstrated a similar time-effect profile: phosphorylated p38 levels began to rise at 6–8 h, peaked at 48–60 h, and later began to decline. Once more, SB203580 inhibited the increases in p38 phosphorylation that were initially induced by the aqueous extracts [22].

**Figure 3:**
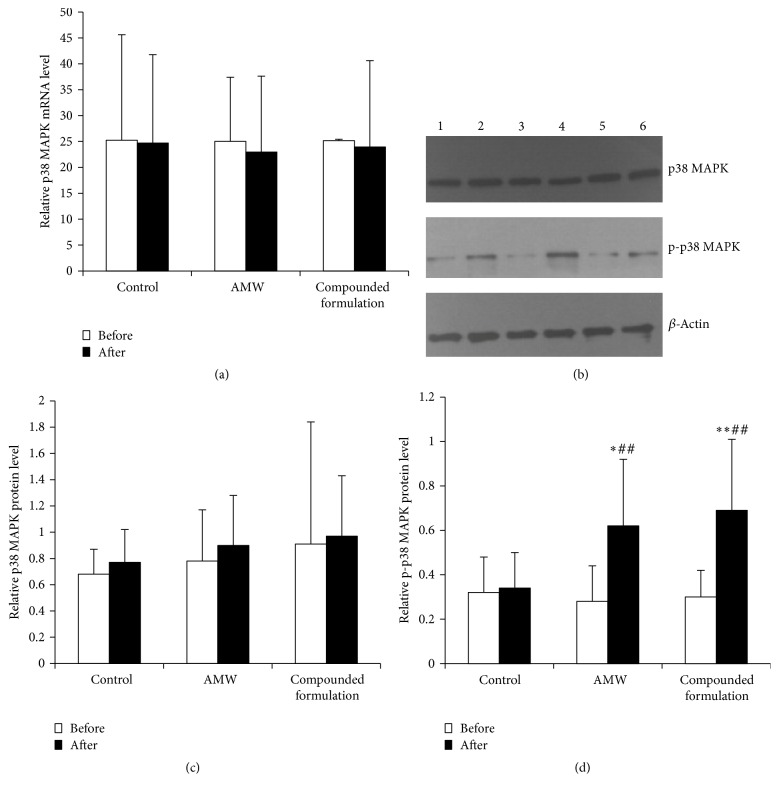
Changes in p38 MAPK expression and activation before and after treatment of pediatric *β*-thalassemia. (a) Changes in mRNA levels. (b) Representative images of Western blot analyses. Graphs of blot quantification data pertaining to total p38 MAPK and phosphorylated p38 MAPK (p-p38 MAPK) levels are shown in (c) and (d), respectively. ^*∗*^ *p* < 0.05 and ^*∗∗*^ *p* < 0.01 compared with the corresponding control; ^##^ *p* < 0.01 compared with before treatment of the same group.

Thus, we measured total p38 MAPK (t-p38) and phosphorylated p38 MAPK (p-p38) levels in nucleated red blood cell-enriched mononuclear cells collected in AMW-, compounded formulation-, or placebo-treated patients. We observed that t-p38 mRNA (Figure 3(a), *p* = 0.999 and 0.965 before and after treatment) and protein (Figures 3(b) and 3(c), *p* = 0.554 and 0.476 before and after treatment) levels were not significantly different between the groups. However, p-p38 protein levels were comparable in all three groups (Figure 3(d); *p* = 0.813) prior to treatment, and protein levels were significantly different after treatment (Figure 3(d); *p* = 0.013). ANOVA analysis and subsequent LSD analysis indicated that p-p38 levels were significantly higher after AMW and compounded formulation treatments compared to placebo treatment. Compared with p-p38 protein levels that were measured before treatment, the compounded formulation and AMW groups expressed 2.3- and 2.2-fold more protein, respectively, after treatment (Figure 3). However, differences between these treatment groups were not significantly different.

We previously reported clinical outcomes pertaining to AMW and compounded formulation treatments [14]. Following 12 weeks of treatment, pediatric patients with *β*-thalassemia had increased fetal Hb, erythrocytes, and mean corpuscular Hb and reticulocytes. Furthermore, no adverse effects were observed with respect to leucocytes, neutrophils, platelets, or hepatic and renal function. The treatment was effective in 64% and 62% of the children with *β*-thalassemia in the compounded formulation and AMW groups, respectively. These outcomes suggest that AMW and the compounded formulation are effective and safe. Moreover, increases in Hb and HbF levels were observed in AMW- and compounded formulation-treated patients, indicating that both treatments may be effective and safe in relation to HbF induction in patients.

Traditional Chinese herbal medicine (TCM) is believed to cause relatively few side effects. However, current reports pertaining to the use of TCM in relation to *β*-thalassemia are complicated and the identification of a single *γ*-globin inducer from TCM preparations is difficult. We previously reported that AMW and an associated compounded formulation for pediatric *β*-thalassemia can significantly improve hematological parameters [14, 16], resulting in elevated HbF levels in patients treated for twelve weeks with AMW or the compounded formulation. Compared with butyrate administration, induction was lasting longer and did not inhibit erythroid cells. Thalassemia is a hematological disorder and several TCM preparations have been reported to improve hematological parameters in anemic patients [17]. More specifically,* Plastrum testudinis* has been shown to induce *γ*-globin expression in erythroid cells (unpublished data).

Based on TCM theory and effects observed* in vitro* and* in vivo*, we developed an AMW formulation and a combined AMW, CPG, and TPG formulation for *β*-thalassemia treatment. As part of this study, we report that treatment with AMW or the compounded formulation for 12 weeks significantly affected G*γ*-globin mRNA levels and increased phosphorylated p38 MAPK levels with no concomitant changes to total p38 MAPK mRNA and protein levels. These results suggest that AMW and the compounded formulation are*γ-globin* gene-inducing reagents. However, because TCM targets multiple receptors, definitive mechanisms of action are often difficult to identify. Larger studies are required to confirm our preliminary results regarding *γ*-globin gene induction and improvements in relation to the occurrence of anemia. In addition, we believe that additional factors including iron load, *β*-thalassemia complications, such as hepatosplenomegaly, heart failure, delayed growth and development, and long-term safety, dose optimization, and treatment regimens pertaining to TCM are also worth exploring. Thus, treatment with AMW and the compounded formulation can induce G*γ*-globin gene expression* in vivo.* This induction is likely to stimulate HbF synthesis and enhance total hemoglobin levels in patients with *β*-thalassemia. Furthermore, independent studies performed by our group and another group demonstrated that the agents used in the treatment regimens used in the current study do not elicit these HbF stimulation phenomena through cytotoxic mechanisms [21, 31]. Thus, we believe that HbF synthesis stimulation may occur via the p38 MAPK signaling pathway.

## References

[1] Muncie H. L., Campbell J. S. (2009). Alpha and beta thalassemia. *American Family Physician*.

[2] Xiang Y., Yu J.-W., Cheng Y.-B. (2013). Study on composing prescription laws of treating aplastic anemia by Chinese medicine using applying data mining technique. *Zhongguo Zhong Xi Yi Jie He Za Zhi*.

[3] Rund D., Rachmilewitz E. (2005). *β*-thalassemia. *The New England Journal of Medicine*.

[4] Schrier S. L. (1997). Pathobiology of thalassemic erythrocytes. *Current Opinion in Hematology*.

[5] Modell B., Darlison M. (2008). Global epidemiology of haemoglobin disorders and derived service indicators. *Bulletin of the World Health Organization*.

[6] Walters M. C. (2010). Gene therapy and bone marrow transplantation for thalassemia: changing of the guard?. *Molecular Therapy*.

[7] Weatherall D. J. (2005). The challenge of thalassemia for the developing countries. *Annals of the New York Academy of Sciences*.

[8] Banan M. (2013). Hydroxyurea treatment in *β*-thalassemia patients: to respond or not to respond?. *Annals of Hematology*.

[9] Bohara V. V., Ray S., Chakrabarti P., Ray S. S., Nath U. K., Chaudhuri U. (2014). Optimizing the dose of hydroxyurea therapy for patients with *β*-thalassemia intermedia (Hb E-*β*-thalassemia): a single center study from eastern India. *Hemoglobin*.

[10] Kosaryan M., Zafari M., Alipur A., Hedayatizadeh-Omran A. (2014). The effect and side effect of hydroxyurea therapy on patients with *β*-thalassemia: a systematic review to December 2012. *Hemoglobin*.

[11] Bianchi N., Zuccato C., Lampronti I., Borgatti M., Gambari R. (2009). Fetal hemoglobin inducers from the natural world: a novel approach for identification of drugs for the treatment of *β*-thalassemia and sickle-cell anemia. *Evidence-Based Complementary and Alternative Medicine*.

[12] Lowrey C. H., Nienhuis A. W. (1993). Brief report: treatment with azacitidine of patients with end-stage *β*- thalassemia. *The New England Journal of Medicine*.

[13] Zhu X., Zhu B. (2001). Effect of Astragalus membranaceus injection on megakaryocyte hematopoiesis in anemic mice. *Hua Xi Yi Ke Da Xue Xue Bao*.

[14] Lu Z.-M., Qian X.-H., Chen Z.-W., Zhang C.-H., Guo L.-S., Chen J. (2012). Prospective clinical study of radix astragali and its compound prescription for treatment of beta-thalassemia in children. *Zhongguo Dang Dai Er Ke Za Zhi*.

[15] Zhang Y., Ye B.-D., Qian L.-L. (2015). Treatment of myelodysplastic syndrome by hematopoietic stem cell transplantation combined with Chinese medical syndrome typing: a clinical study. *Zhongguo Zhong Xi Yi Jie He Za Zhi*.

[16] Yang M., Qian X.-H., Zhao D.-H., Fu S.-Z. (2010). Effects of Astragalus polysaccharide on the erythroid lineage and microarray analysis in K562 cells. *Journal of Ethnopharmacology*.

[17] Linn Y.-C., Lu J., Lim L.-C., Sun H., Sun J., Zhou Y. (2011). Traditional Chinese herbal medicine in the supportive management of patients with chronic cytopaenic marrow diseases—a phase I/II clinical study. *Complementary Therapies in Clinical Practice*.

[18] Liu Y.-M., Wu Z.-K., Chai L.-M. (2007). Effect on expression of mice alpha-hemoglobin stabilizing protein in different developmental stages treated with Yisui Shengxue granules. *Zhongguo Zhong Yao Za Zhi*.

[19] Qian X., Chen J., Zhao D., Guo L., Qian X. (2013). Plastrum testudinis induces *γ*-globin gene expression through epigenetic histone modifications within the *γ*-globin gene promoter via activation of the p38 MAPK signaling pathway. *International Journal of Molecular Medicine*.

[20] Chang M. S., Kim D. R., Ko E. B. (2009). Treatment with astragali radix and angelicae radix enhances erythropoietin gene expression in the cyclophosphamide-induced anemic rat. *Journal of Medicinal Food*.

[21] Guo Z.-M., Li H.-J., Qian X.-H. (2008). *γ*-globin synthesis in K562 cells induced with *Tortois plastron*, *Astragali*, *Salviae miltiorrhizae* and *Codonopsis pilosulae*. *Zhongguo Shi Yan Xue Ye Xue Za Zhi*.

[22] Zhao D. (2008). *Role of p38 MAP kinase activation in radix astragali-mediated γ-globin gene expression [M.S. thesis]*.

[23] Zhang Z. N., Shen D. (2007). *Criteria for Diagnosis and Treatment of Hematopathy*.

[24] Kwon K. H., Jeon Y. J., Hwang H. S. (2007). A high yield of fetal nucleated red blood cells isolated using optimal osmolality and a double-density gradient system. *Prenatal Diagnosis*.

[25] Martín-Blanco E. (2000). p38 MAPK signalling cascades: ancient roles new functions. *BioEssays*.

[26] Čokić V. P., Smith R. D., Biancotto A., Noguchi C. T., Puri R. K., Schechter A. N. (2013). Globin gene expression in correlation with G protein-related genes during erythroid differentiation. *BMC Genomics*.

[27] Chou Y.-C., Chen R.-L., Lai Z.-S., Song J.-S., Chao Y.-S., Shen C.-K. J. (2015). Pharmacological induction of human fetal globin gene in hydroxyurea-resistant primary adult erythroid cells. *Molecular and Cellular Biology*.

[28] Steinbrech D. S., Mehrara B. J., Saadeh P. B. (2000). VEGF expression in an osteoblast-like cell line is regulated by a hypoxia response mechanism. *American Journal of Physiology—Cell Physiology*.

[29] Ramakrishnan V., Pace B. S. (2011). Regulation of *γ*-globin gene expression involves signaling through the p38 MAPK/CREB1 pathway. *Blood Cells, Molecules, & Diseases*.

[30] Pace B. S., Qian X.-H., Sangerman J. (2003). p38 MAP kinase activation mediates *γ*-globin gene induction in erythroid progenitors. *Experimental Hematology*.

[31] Yang M., Zhao D. H., Qian X. H. (2008). Effects of the main extracts of *Astragalus membranaceus* on inducing the erythroid differentiation of K562 cells. *Medical Journal of Chinese People's Liberation Army*.

